# Cultural Predictors of Resilience in a Multinational Sample of Trauma Survivors

**DOI:** 10.3389/fpsyg.2019.00131

**Published:** 2019-02-05

**Authors:** Sumithra S. Raghavan, Priyadharshiny Sandanapitchai

**Affiliations:** Department of Psychology, William Paterson University, Wayne, NJ, United States

**Keywords:** cross-cultural, trauma, resilience, PTSD, cultural differences

## Abstract

The present study aims to fill a gap in the study of resilience to trauma by examining resilience in a culturally diverse population. Approximately 70% of adults across the globe experience at least one traumatic event in their lifetime, yet resilience is a common response trajectory. This pilot study explored reactions to trauma and psychological resilience in an international sample of trauma-exposed participants. Participants were recruited online using the Amazon Mechanical Turk software and after completing an informed consent, were determined eligible to participate if they endorsed experiencing at least one traumatic event. Eligible participants then completed The Stressful Life Events Questionnaire, Brief Resiliency Scale, Ego Resiliency Scale, Posttraumatic Stress Disorder Checklist-Civilian Version, Brief Religious Coping Scale, and Multigroup Ethnic Identity Measure. The final sample included 200 trauma exposed adults from nineteen different countries worldwide, with a majority hailing from the United States, India, Sri Lanka and the Philippines. Results revealed that Asian participants scored significantly higher on resilience scales and endorsed higher levels of spiritually focused coping than other subgroups. Multivariate analyses revealed that these differences in resilience remained significant even after controlling for sense of ethnic identity and spiritual coping, suggesting that there may be culturally specific predictors of resilience within the Asian subgroup. Understanding variations in resilience will aid in developing culturally tailored interventions and pursuing a strengths-based approach to recovery from trauma. Limitations and implications are discussed.

## Introduction

Research indicates that approximately 70% of adults globally (Benjet et al., 2016) and 89.7% of adults in the United States (Kilpatrick et al., 2013) experience at least one traumatic event during their lifetimes. The negative consequences of trauma exposure are well documented, including psychological (Turner and Lloyd, 1995) and somatic symptoms (Chester and Holtan, 1992). Despite this data, the prevalence of PTSD in United States population is only 6.8% (Gradus, 2013), and is similarly low worldwide (Kessler et al., 2017). While rates may reflect underreporting, it is also likely that many are able to display resilience (Bonanno et al., 2011).

Decades of research reveal evolving definitions of resilience to trauma. Early scholars categorized it as an internal construct, relating to traits such as self-esteem and goal-orientation (Block and Block, 1980; Rutter, 1985). Groundbreaking work by Werner et al. (1971) challenged this notion by demonstrating that among children who endured multiple traumas, one third were thriving in adulthood due largely to external supports. Werner’s results pointed to the value of situational and contextual factors in facilitating resilience. It is worth noting that resilience is distinct from its related construct of posttraumatic growth (PTG; Tedeschi and Calhoun, 2004) which refers to one’s ability to thrive and improve, particularly in interpersonal relationships, after exposure to trauma. Resilience, in contrast, focuses on one’s ability to return to previous levels of functioning (Masten et al., 1990).

Definitions of resilience should incorporate culture, which includes an individual’s social, political, interpersonal and familial contexts, and acts as a lens through which people view their world (Triandis, 1972). Cultural values and beliefs impact both an individual’s interpretation of a traumatic event as well as his/her reaction to it (Kalmanowitz and Ho, 2017). Ungar (2017) speaks to the idea that much of the research on resilience is so steeped in a Western-centric value system that emphasizes individual qualities that it fails to identify important protective factors that may relate to culture. He adds that by incorporating the concept of culture, researchers can fundamentally change assumptions about functionally adaptive behavior and question the individualistic interpretation of coping (Ungar, 2013).

A study by Afana et al. (2010) involved ethnographic interviews with key informants in the Gaza Strip. The researchers theorized that social constructions and interpretations of trauma, informed by cultural views, could provide a valuable window into posttraumatic reactions. Interview data supported their hypothesis: across eight respondents, researchers identified linguistic idioms of distress that described clusters of psychological and somatic reactions as well as methods of coping with their trauma exposure. They concluded that these culturally specific reactions to trauma did not map against standard Western diagnostic criteria but provided a window into their construction of resilience.

Regarding specific dimensions of culture, research points to the protective impact of spiritual or religious beliefs (i.e., Pargament et al., 2011) as well as strong affiliation with ethnic identity (i.e., Han et al., 2016). For example, recent research by Veronese et al., 2017 pointed to the protective impact of spirituality and sense of meaning amongst trauma exposed Palestinian aid workers in the Gaza Strip. In fact, the researchers demonstrated that participants with the highest levels of trauma exposure could experience resilience through spiritual and religious affiliations.

Similarly, affiliation with one’s ethnic identity can have a powerful impact on mental health, especially for marginalized groups (Han et al., 2016). Wexler’s (2014) mixed-methods examination of indigenous adults in Canada revealed that elders believed that strong identification with their Native heritage would allow them to resist the pressures of acculturation that arose from colonization, thus ethnic identification became a source of strength after trauma.

Despite calls to include dimensions of culture in studying resilience, researchers such as Feldman and Masalha (2007) argue that it remains an under explored topic. The present study aims to fill this gap by examining cultural contributors to resilience in a multinational sample of trauma survivors. We hypothesized that resilience scores would vary between groups, as would sense of ethnic identity and use of spiritual coping. We further anticipated that ethnicity would predict resilience scores even after accounting for the impact of ethnic identity and use of spiritual coping.

## Methods

### Participants and Procedure

Participants were a multinational sample of 200 trauma-exposed adults (aged 18 or over) recruited through Amazon Mechanical Turk Program (MTurk), a popular platform for human subject research in the social sciences. Detailed information regarding participant demographics is described in the Results section. Regarding the data collection tool, research indicates that MTurk produces high quality data from diverse samples with equal or greater reliability than data obtained through traditional methods (Buhrmester et al., 2011). After completing an informed consent, participants who reported at least one traumatic event were deemed eligible for the study and were invited to complete the remaining study measures regarding trauma and resilience. Given that study participants completed this survey online, there was no identifying information connected to study data and participant privacy was protected. The study was carried out in accordance with the principles of the American Psychological Association and all study materials were approved by the William Paterson University Institutional Review Board (IRB).

### Measures

#### Demographic Questionnaire

Participants were asked about their trauma exposure, spirituality and ethnic identity, along with standard demographic questions including proficiency with English language.

#### Stressful Life Events Screening Questionnaire-Revised (SLESQ-R)

The SLESQ-R is a 13-item self-report measure that asks clients to describe lifetime trauma exposures that could qualify as Criterion A for a PTSD diagnosis (Ruiz-Párraga and López-Martínez, 2014). The SLESQ-R has demonstrated sound reliability and validity and has been used with diverse samples (López-Martínez et al., 2014). In the current sample, Cronbach’s α = 0.86.

#### Psychopathic Personality Inventory Deviant Responding Subscale (PPI-DRS)

Psychopathic personality inventory deviant responding subscale contains 10 questions to identify aspects of psychopathy in non-clinical population (Douglas et al., 2006). This scale was used to detect random responding of the participants and has been used in previous research with MTurk samples (see Crowe and McKay, 2016). Participants who score more than 5 were considered to be “random responders” and eliminated from the study. Cronbach’s alpha for the PPI subscales range from 0.70 to 0.89 and test-retest reliabilities ranges from 0.82 to 0.94 (Poythress et al., 1998). In the present study, Cronbach’s α = 0.74.

#### Posttraumatic Stress Disorder Checklist–Civilian Version (PCL-C)

Posttraumatic stress disorder checklist–civilian version is a 20-item self-report measure, corresponding to the symptoms of PTSD in DSM 5 (Blevins et al., 2015). Respondents endorse the extent to which they are bothered by symptoms ranging from zero (Not at all) to four (extremely). The PCL-C may also be used to diagnose PTSD if participants score three or higher on one item from questions one though five, three items from questions six through twelve, and two items from questions thirteen through seventeen. The PCL-C is a commonly used measure with high validity and reliability (Gore et al., 2013), and current alpha levels are 0.96.

#### Ego Resiliency Scale (ERS)

Ego resiliency scale is a14-item scale which measures the ability of an individual to adapt to changing environmental demands in face of adversity (Farkas and Orosz, 2015). Respondents rate statements on a scale from one (does not apply at all) to four (applies very strongly). The scale demonstrated reliability and validity (Resnick et al., 2011) with Cronbach’s α for the present sample equal to 0.95.

#### Brief Resiliency Scale (BRS)

Brief resiliency scale is a 6-item, 5-point scale which assesses the ability to bounce back from stress. BRS has a high internal consistency (Shakespeare-Finch and Daley, 2017) and has been used widely in resilience research, with higher scores correlating with higher self-reported resilience (Sánchez-Álvarez et al., 2016). In the present sample, α = 0.88.

#### Brief Religious Coping Activity Scale (Brief RCOPE)

The 14-item Brief RCOPE assesses the use of religious coping methods during adverse life events. The scale incorporates both positive religious coping (forgiveness, spiritual connection and support) and negative religious coping (Questioning god’s power and love, lack of devotion). The items are scored from one being *Not at all* and four being *A great deal* (Cotton et al., 2006). The scale has strong validity and internal consistency (Pargament et al., 2004) and current alpha levels are 0.96.

#### Multigroup Ethnic Identity Measure (MEIM)

The MEIM (Phinney, 1992) is a brief measure assessing elements of ethnic identity that can be used across multiple groups. Respondents rate on a one to four scale the degree to which they agree with a series of statements regarding the role of ethnicity in their identity and daily life. The measure has sound reliability and validity and has been used with a wide range of samples, with current alpha levels measure 0.88.

### Data Analysis

After assessing normality, univariate analyses of variance (ANOVAs) were performed to examine differences between groups on resilience scores, ethnic identity and spiritual coping. Subsequently, a multivariate analysis of covariance (MANCOVA) was conducted to examine whether the ethnicity variable impacts resilience scores even after accounting for the impact of spirituality and ethnic identity.

## Results

A total of 217 participants completed the full online battery of questionnaires. There was a small amount of missing data due to skipped questions, and six participants earned a PPI-DR score greater than 5 suggesting unreliable responding. The final sample consisted of 200 participants (68% male and 31.5% female) who completed the online survey. Participants were largely from the United States (48.5%) and Asian countries including India, Sri Lanka and the Philippines (33.5%), but the sample included participants from 19 countries worldwide. Regarding racial and ethnic composition, a majority of the participants identified themselves as non-white including Asians (33.5%), Hispanics (9 %), Blacks (5%), and Multiracial (8%) compared to those who identified as White (44%). In terms of religious affiliation, participants reported to be affiliated with Christianity (27%), Hinduism (25.5%), Judaism (1.5%), Islam (1%), and others (4.0%). Table 1 below shows a summary of additional participant demographics. Among those participants who reported that English was not their native language, 97% (*n* = 45) indicated that they speak, read, or write English almost daily. While we did not obtain data regarding household income, participants were asked two questions regarding finances: first, whether they considered themselves financially secure, stable or unstable (described in Table 1) and second, how frequently they worry about finances on a five-point scale, with five signifying constant worry. Participants reported moderate worry about finances (*M* = 3.5, *SD* = 1.15).

**Table 1:** Demographic information.

	N	Percentage
**Age**		
18–24	31	15.5
25–35	128	64.0
36–50	33	16.5
51–64	8	4.0
**Education**		
Primary school	1	0.5
High school graduate or its equivalent (i.e., GED)	53	26.5
College graduate	124	62.0
Advanced/professional degree	22	11.0
**Employed**		
Yes	148	74.0
No	51	25.5
**Marital status**		
Single	88	44.0
Married	72	36.0
In a relationship	37	18.5
Others	3	1.5
**English: native language**		
Yes	151	75.5
No	48	24.0
**Financial status**		
Secure (able to afford basic necessities + luxuries)	58	29
Stable (able to afford basic necessities)	97	48.5
Unstable (struggling to make ends meet)	45	22.5


Scores on the SLESQ showed 93% of participants (*n* = 186) reported exposure to more than one traumatic event. The most commonly reported traumatic events were having a close family member, friend or romantic partner die due to accident, homicide or suicide (43.5%, *n* = 87) or being in a life-threatening accident (42%, *n* = 84). Additionally, 60% (*n* = 120) met criteria for a diagnosis of PTSD based upon the scores on the PCL-C, although this was not verified with a clinical interview and results should be interpreted with caution. Participants who met criteria for PTSD did not differ significantly on demographic variables compared to those who did not meet criteria, however, those with PTSD did evidence significantly lower scores on the BRS [*t*(2,191) = 5.87, *p* < 0.01].

Despite elevated levels of trauma, participants also endorsed high levels of resilience, strong affiliation with ethnic identity, and use of spiritual coping tools. Average MEIM scores were 2.27 (*SD* = 0.69). Scores on the five-point BRS were 3.25 (*SD* = 0.52) and on the four-point ERS, 2.92 (*SD* = 0.52). On the positive religious coping scale, participants’ total average score was 2.12, which is slightly above the average score of Brief R-Cope subscale.

One-way analyses of variance indicated significant differences between ethnic groups on resilience, ethnic identity and spiritual coping scores (see Table 2). *Post hoc* Games-Howell tests showed that participants who identified as Asian scored significantly higher on both resilience scales and on the positive religious coping scale in comparison to other participants.

**Table 2:** Assessing normality, univariate analyses of variance of resilience scores, religious coping and ethnic identity by ethnicity.

Scales		Df	*η*	*F*	*P*
BRS score	Between groups	4	0.01	0.42	0.00^∗^
	Within groups	183	0.90		
	Total	187			
ERS score	Between groups	4	0.10	7.6	0.00^∗^
	Within groups	186	0.90		
	Total	190			
Positive religious coping	Between groups	4	0.10	5.3	0.001^∗^
	Within groups	181	0.90		
	Total	185			
MEIM score	Between groups	4	0.07	3.78	0.006^∗^
	Within groups	185			
	Total	189			


*^∗^ = denotes significance at p < 0.05 level.*

All significant findings were entering into a general linear model and multivariate analyses of co-variance (MANCOVA) were performed to examine whether ethnicity impacted scores on resilience scales even after accounting for the impact of coping and ethnic identity. Results were indeed statistically significant and revealed that there were differences in resilience scores between ethnic groups even after controlling for coping and ethnic identity, *F*(8,340) = 1.83, *p* < 0.05, Pillai’s Trace = 0.09, partial η^2^ = 0.0. Additionally, spiritual coping remained a unique contributor to resilience scores, while ethnic identity did not, *F*(8,340) = 24.22, *p* < 0.01, Pillai’s Trace = 0.22, partial η^2^ = 0.01.

## Discussion

Results indicate that among this multinational sample, there are high levels of trauma exposure and posttraumatic symptoms, along with high levels of reported resilience, with significant differences between groups. Specifically, participants who identified as Asian or South Asian scored significantly higher on resilience scores, affiliation with ethnic identity, and use of positive spiritual coping than other groups. These findings are consistent with resilience literature in specific cultural groups that points to use of religious or spiritual coping (Reinert et al., 2015), as well as pride in ethnic identity (Moscardino et al., 2007).

The higher number of Asian participants and elevated rates of trauma bear further exploration. While we sought a broad-based international sample, we received many responses from participants of Asian origin. This may be due to the fact that the primary investigators are of South Asian origin themselves and participants may have recognized South Asian names and felt more comfortable completing survey. Among the Asian subgroup specifically, participants most commonly reported traumas were losing a family member or close friend to a serious accident, homicide or suicide (54%, *n* = 36), being in a life-threatening accident (42%, *n* = 28) and having a life-threatening illness (39%, *n* = 26). The Asian sample was comprised primarily of participants from India, Sri Lanka, and the Philippines, all areas that have suffered significant natural disasters, civil and social unrest. However, it is interesting to note that similar trauma was reported in the White sample, with the exception of having a life-threatening illness. That said, participants of Asian origin did endorse higher rates of all traumas and high scores on resilience measures than the large White subgroup, and future studies may benefit from comparing these two specific samples.

Additionally, even after accounting for spiritual coping and ethnic identity, results remained significant, suggesting that additional components of the ethnicity construct are impacting resilience scores. This is also conversant with the small but growing body of work focusing on cultural contributors to resilience (i.e., Marie et al., 2016; Afana et al., 2018). Given that, future research should focus on identifying those additional components of the ethnicity construct and teasing out their impact on psychological resilience.

A total of 44 participants (*n* = 22%) identified as hailing from fourteen countries outside of Asia and the United States, with the largest group (*n* = 7) being form Venezuela. As such, the sample sizes are incredibly small and preclude us from drawing meaningful comparisons to our larger White and Asian samples. It is noteworthy that this multinational subgroup also endorsed the same commonly reported traumatic events endorsed by the Asian sample and did not differ from the Asian or White sample on demographic variables such as financial status or education level. Our call for participation was open to respondents all over the world, and future research may benefit from focusing on several specific countries to recruit even samples, rather than broadly drawing an international sample.

One of the main limitations of the study was the use of Westernized concept of “resilience” in a non-Western sample. While the measures employed have been used with diverse populations, we did not complete thorough invariance testing to ensure psychometric equivalence. That said, alpha levels within our sample were all above 0.80 and 97% of our sample reported speaking and writing in English on a daily basis. Future studies would benefit from a mixed-methods exploration and the inclusion of narrative data. In addition, the choice of ethnic identity and religiosity were used to operationalize culture, and there are many other variables that comprise the “ethnicity” variable that warrant further exploration. More specifically, our sample overall reported high levels of resilience, which may relate to demographic variables of education and economic class rather than ethnicity. A majority of the sample reported completing college and described themselves as financially stable, two factors that data support as being predictive of resilience. Lastly, the differing sample sizes between groups may have impacted the analyses. As previously mentioned, we were surprised at the number of Asian/South Asian participants, which comprised a fairly large subgroup in the sample.

Despite these limitations, this small-scale pilot study may be used to begin a conversation about understanding cultural contributors to resilience in a traumatized sample. Results confirmed that adults in this sample are exposed to high levels of trauma and suffering from distress yet report the ability to function and bounce back from these experiences. These findings have interesting implications for clinical work, including highlighting the need for culturally competent practitioners and the incorporation of indigenous values and practices into clinical work. More specifically, trauma survivors may benefit from culturally tailored interventions and opportunities to draw upon cultural strengths in the healing process.

## Ethics Statement

This study was carried out in accordance with the recommendations of William Paterson University Institutional Review Board with written informed consent from all subjects. All subjects gave written informed consent in accordance with the Declaration of Helsinki. The protocol was approved by the Institutional Review Board of William Paterson University.

## Author Contributions

SR and PS worked together on all aspects of the research process and publication. SR wrote the introduction, results and discussion while PS contributed significantly to the methods, references and final editing. PS was responsible for data cleaning and database management. PS is a graduate student author.

## Conflict of Interest Statement

The authors declare that the research was conducted in the absence of any commercial or financial relationships that could be construed as a potential conflict of interest.
